# Religion Does Matter for Climate Change Attitudes and Behavior

**DOI:** 10.1371/journal.pone.0134868

**Published:** 2015-08-06

**Authors:** Mark Morrison, Roderick Duncan, Kevin Parton

**Affiliations:** 1 School of Management and Marketing, Charles Sturt University, Bathurst, New South Wales, Australia; 2 Institute for Land, Water and Society, Charles Sturt University, Bathurst, New South Wales, Australia; 3 School of Accounting and Finance, Charles Sturt University, Bathurst, New South Wales, Australia; University of Washington, UNITED STATES

## Abstract

Little research has focused on the relationship between religion and climate change attitudes and behavior. Further, while there have been some studies examining the relationship between environmental attitudes and religion, most are focused on Christian denominations and secularism, and few have examined other religions such as Buddhism. Using an online survey of 1,927 Australians we examined links between membership of four religious groupings (Buddhists, Christian literalists and non-literalists, and Secularists) and climate change attitudes and behaviors. Differences were found across religious groups in terms of their belief in: (a) human induced climate change, (b) the level of consensus among scientists, (c) their own efficacy, and (d) the need for policy responses. We show, using ordinal regression, that religion explains these differences even after taking into account socio-demographic factors, knowledge and environmental attitude, including belief in man’s dominion over nature. Differences in attitude and behavior between these religious groups suggest the importance of engaging denominations to encourage change in attitudes and behavior among their members.

## Introduction

Views on climate change and policy relating to climate change in the Australian population are extremely diverse [1]. In forming their views, people are influenced by many factors, including both situational variables and their own socio-economic and socio-political status [2]. In this paper we focus on religious affiliation as a potential determinant of attitudes to climate change and climate change policy.

Research conducted, principally in the United States (US) and Europe, has indicated that religious affiliation is a key factor to take into account in developing climate change policy and designing messages about policy [3]. Based on an examination of teachings of nine major religions, covering issues such as other-person centeredness and environmental stewardship, Posas [4] argued strongly that religions from Bahá’í to Buddhism and from Islam to Christianity should be able to influence their members to bring an ethical dimension which is sympathetic to climate change policy. White [5] contended that there is a link between a Judeo-Christian perspective and a desire for dominion over nature, and that it was this dominion attitude which explained the scale of environmental destruction in the modern world. This dominion perspective has its basis in Genesis 1:26, ‘Then God said, “Let us make mankind in our image, in our likeness, so that they may rule over the fish in the sea and the birds in the sky, over the livestock and all the wild animals, and over all the creatures that move along the ground”‘. In the US, a number of studies have revealed that this perspective manifests itself in a conservative Christianity effect, under which those who have a strong literal interpretation of the bible have a lower concern about the environment and a stronger belief in their own efficacy in controlling outcomes (e.g. [6, 7]). This effect has a considerable influence when measuring the overall level of denial of climate change and the perceived need for policy. While clearly embedded in this discussion are the theological positions of the major religions, our focus throughout this paper is on climate change attitudes and behavior of those claiming affiliation with particular religious groups.

The literature also reveals that there are important differences between countries in the influence that religious affiliation has on ways of looking at climate change issues [8]. This fact points to a need to consider the religious issues within a particular country context. There has been little attention given to these issues in Australia, and consequently one objective of the current paper is to examine how religious make-up affects attitudes and behavior in relation to climate change and climate change policy. Moreover, there are important features of the Australian situation that suggest that it is a country worthy of study. Dramatic societal change will be required there to achieve substantial carbon emission reductions. Although it is only a moderate carbon emitter in aggregate terms (ranked 17^th^ in the world), in per capita terms it is the world’s second highest emitter [9]. Also it is particularly dependent on the coal economy and will require considerable structural change to progress towards a low-carbon economy. These considerations mean that in future Australia could be an indicator of global progress on combating climate change.

We conducted an online survey of 1,927 Australians, designed to identify household segments (groups of households with similar behaviors and attitudes about climate change) based on the methodology developed by Maibach et al. [10], as well as highlight differences in Australia between different religious groups. We examined responses across four religious groupings (Atheist/Agnostic/No Religion, Buddhist, Christian non-literalists and Christian literalists). We found substantive effects on climate change beliefs based on religious affiliation. For the non-religious grouping (Atheist/Agnostic/No Religion) the differences in beliefs can be explained by either socio-demographics, environmental attitudes or environmental knowledge. However for the other groupings (Buddhist, Christian literalists and Christian non-literalists) the effects cannot be explained by these variables alone.

Empirical work has shown that there are distinctly different Christian environmental attitudes [3]. Within Christian circles there are contrasting themes of “anthropomorphic dominance” and “stewardship of nature”. Hand and van Liere [6] showed that in the US, members of denominations such as Baptists and Mormons were more likely to adhere to the first, while Episcopalians and Methodists the second. Moreover, it is these world views that seem to determine whether an individual would support environmental policy. In an extension of this research, an examination of Christian liberalism in the US revealed that support for spending on the environment correlates with an image of God that is gracious (i.e. benevolent and not judgmental) and with being Catholic [11, 12, 13]; and that a rigid political and religious view is related to a lack of environmental concern [14, 15]. However, there was no significant difference between Judeo-Christians in general and other religions in regard to environmental concerns. The Forum on Religion and Ecology at Yale provides a comprehensive bibliography of the literature on the environmental stance of the major religions [16].

Focusing more closely on American evangelical Christians, Smith and Leiserowitz [3] showed that within this group there is a diversity of views on climate change and that this diversity is associated with “affect based value orientations, ideologies and worldviews” (p.8) rather than religion. Nevertheless, even though a majority believes in climate change, compared with non-evangelicals, American evangelicals are less likely to believe that (a) climate change is occurring, (b) human activity is the cause, and (c) scientists think that climate change is occurring.

Greeley [14] and Kanagy and Nelson [17] highlighted the problem of focusing only on religious variables to explain variations in concern for the environment. Their studies showed that when cultural, social and demographic influences were taken into account, significant relationships between Christian perspective and dominion over nature were no longer apparent. These authors identified, therefore, a complex relationship between religious affiliation, socio-demographics and environmental concern. Taking into account this complexity leads to the conclusion that “religious individuals—even those identified as conservative—are no less likely than non-religious individuals to identify themselves as environmentalists” [17].

Hence, this line of research effectively concluded that the association between a Christian perspective and dominion over nature was spurious [14, 17]. The apparent relationship may rather be a reflection of political conservatism, or some other socio-demographic variable [18, 19]. However, a thorn remained in the side of this position. One consistent result from US studies is in accord with White’s thesis, and shows that those adhering more closely to a literal biblical viewpoint are less likely to support pro-environmental action [3, 7]. In addition, Guth et al. [7] showed that US “secularists” generally support environmental policy, while Catholics are in the middle ground between the above extremes. Taken together, these results support the contention that a more appropriate conclusion is that religious factors are indirectly causal rather than spurious. Furthermore, a key variable emerging from the literature on Christian belief was literal interpretation of the bible. The large number of Christians attached to evangelical churches in the US, likely to hold more literal views, is an important issue in overall public opinion. Australia has a smaller proportion of Christians who identify as evangelicals. Nevertheless, given the differences observed in the US, it did seem worthwhile to include a focus in our work on the distinction in climate change attitudes and behavior between literal and non-literal Christians in Australia.

More recently Truelove and Joireman [20], developed two scales: a Christian orthodoxy scale, and a biblical literal scale. Their thesis was that there could be either a “social-altruistic” effect that would have Christians supportive of environmental policy, or a “lower-knowledge-of-the-biosphere” effect that would produce the reverse effect. Then, from survey results, they discovered that Christian orthodoxy and biblical literalism were inversely associated with all measures of environmental behavior, and that the lower-knowledge-of-the-biosphere effect was dominant. Similarly, the impact of respondents’ scientific knowledge has also been shown to be critical in other studies in Germany [21], Sweden [22] and Britain [23]. Thus a need to take into account further mediating variables, altruism and knowledge, when assessing the relationship between religion and attitudes towards the environment has been suggested by these studies.

Sherkat and Ellison [24] attempted to reconcile the previous empirical work on the relationship between Christian beliefs, political beliefs, socio-demographics and environmental attitudes. They used structural equation modelling based on survey data from the United States to estimate the effect of various beliefs and socio-demographic variables on environmental orientation. Political and private environmental activism was found to be significantly negatively associated with political conservatism, biblical inerrancy (political activism only), conservative Protestantism, rurality and southern (US) location, and positively associated with belief in problem seriousness, stewardship, and education level. Sherkat and Ellison [24] concluded that: “Future studies investigating the connections between religious factors and a host of other political concerns would benefit from adapting a more comprehensive view of religious influences, and attending to nuances of political beliefs and connections.”

For Buddhism the relationship between environmental concern and religious affiliation is a complex one. White [5] had contrasted the dominion over nature view which he felt lay at the base of Judeo-Christian faiths with the “very nearly the mirror image” view on the human-nature relationship of Buddhism [5]. Harris [25] challenged this characterization by White and argued instead that the Buddhist goal of detachment includes detachment from the natural environment. The injunction in classical Buddhist texts for monks not to dig in the ground due to the possibility of injuring a worm is driven more, Harris argued, by concern to avoid spiritual pollution for the monk than it is by any inherent value for the natural environment [25]. Other writers [26, 27, 28] have argued that the White interpretation of Buddhist environmental principles is a modern Western creation that bears little resemblance to traditional Buddhist views.

Some support for Harris’s statement was found by Hayes and Marangudakis [23] in their research on environmental attitudes in Britain. They found no difference in dominion over nature attitudes for followers of Abrahamic religions (Jews, Christians and Muslims) as opposed to their category of non-Abrahamic which included Buddhists, Hindus, Sikhs and other faiths with a stated belief in a higher power. Unfortunately their failure to explore possible differences within religions within the non-Abrahamic category makes this a rather limited result. We are aware of no other research that has empirically examined the relationship between Buddhist beliefs and environmental or climate change attitudes.

Despite the research activity proceeding elsewhere, there has been little consideration in Australia of the relationships between climate change and religion [29]. Recent studies have tended to focus on the influence of belief about future temperature increases or the efficacy of policy [1], and the sources of scepticism about climate change [30]. Tranter’s [2] research comes closest to examining climate change and religion, but includes results only for those with no religion and those engaging in spiritual religious practices. It is important that more research on religion is conducted in Australia, because as Tjernstron and Tietenberg [8] show there is considerable difficulty in simply transferring results from one national jurisdiction to another. By comparing survey results from 26 countries, they showed that individual attitudes towards climate change affect the type of climate change policy that different countries introduce. Further, individual attitudes are shaped by how individuals react to the specific consequence of climate change, information, the openness of their society, and by attitudes toward the trustworthiness of government.

Thus the evidence from the literature is that religious affiliation can affect environmental attitudes. However in some cases differences in environmental attitudes across religious groupings may reflect socio-demographic variables, political conservatism or scientific knowledge. Furthermore, it has been demonstrated that there are several distinct environmental worldviews that exist within Christian thought and that some are quite supportive of climate action. Thus, it should be recognized that within religious groupings as well as across religious groupings there is likely to be heterogeneity in perspectives.

This literature directed our research to examine the following five issues: After accounting for other socio-demographic influences and environmental knowledge, are there different attitudes and behavior in relation to climate change depending on religious affiliation? Are members of certain religious affiliations less likely than other members of the community to support climate change action? Does lower knowledge of climate change issues result in lower support for climate change action? Are there broad systems of belief that form the basis of climate change attitudes and behavior? Can differences in climate change attitudes and behavior be explained by the White hypothesis about beliefs in man’s dominion over nature? These issues provided the motivation for the analysis described in the remainder of this paper.

## Materials and Methods

The Charles Sturt University Human Ethics Committee provided ethics approval for the survey work which involved an online questionnaire. By administering this questionnaire, we surveyed 1,927 Australian respondents selected from a panel of respondents provided by the Online Research Unit (ORU). A two-stage probabilistic sampling procedure was used, which involved initial use of random sampling within the sample frame, but then additional random sampling within specific sections of the sample frame to ensure representativeness of the population across gender and age. The final sample excludes incomplete responses and those who rushed the survey (completed in less than 8 minutes).

Comparison of the socio-demographics of the sample and the Australian population confirmed that the sampling procedure ensured that the sample corresponded closely with the population statistics. The mean age in the sample was 47.37 years (population 45.31 years), gender in the sample was 48.95% male (population 48.82%) and gross weekly family income in the sample was $1,432 (population $1,407) [31].

The first stage of our analysis involved identifying climate change segments. We used the same 36 questions in the questionnaire as the Maibach et al. [10] survey. Consistent with the main analysis in the ‘six-America’s study’ [10], we used Latent Class Analysis to derive segments. We identified the same six segments as Maibach et al. that differ in their attitudes and behaviors towards climate change. Thus the method of segmentation is driven by respondents’ attitudes and behavior with respect to climate change. The six segments are: *Alarmed*, *Concerned*, *Cautious*, *Disengaged*, *Doubtful* and *Dismissive*. The *Alarmed* segment are the most concerned about climate change, and the *Dismissive* segment are the least concerned and generally do not believe that it is occurring. A comparison between the segmentation results from Maibach et al.’s [10] study and our segmentation results is presented in Table 1. The key result was that compared with the US, there are fewer in the *Alarmed* and *Concerned* segments in Australia, about the same proportion in the *Doubtful* and *Dismissive* segments, and consequently a much higher proportion in the *Cautious* and *Disengaged* segments. As discussed further in [32], this seemed to indicate that there was much scope to develop majority support for climate policy in Australia by focusing on these middle two groups.

**Table 1:** Segment size in US versus Australia.

	Alarmed	Concerned	Cautious	Disengaged	Doubtful	Dismissive
US (Maibach et al. 2011)	18%	33%	19%	12%	11%	7%
Australia	10.8%	22.5%	26.1%	20.0%	11.3%	9.3%

Source: [32]

Second we examined how segment membership differed across religious affiliations. To do this we included a question about religious affiliation. For those who identified as belonging to one of the Christian denominations, we grouped them according to whether they belonged to a denomination that generally treats the bible as being the literal truth (i.e. believe in biblical inerrancy), and those denominations that do not. Although there is useful work that has been conducted in the US showing the degree to which members of different denominations believe in biblical inerrancy [33, 34, 35], there is less research on this issue in Australia [36, 37]. Consequently, the following classification of literal and non-literal Christian denominations in Australia is based on this literature combined with various publications of the National Church Life Survey [38]. Denominations that were treated as having a literal view of scripture included Baptist, Presbyterian, Church of Christ, Lutheran, Evangelical and Pentocostal churches. Denominations treated as not having a literal view of scripture were: Catholic, Orthodox, Anglican and Uniting (a merger of Methodist, Congregational and many of the Presbyterian churches across Australia in 1977) churches. (A few Anglican dioceses (e.g. Sydney) teach a literal view of scripture. However, as these dioceses make up less than 20% of all dioceses within Australia, Anglicans were treated as being non-literal.) Finally, those religious groups which had fewer than 15 respondents identifying as belonging to them were excluded from further analysis. This had the effect of removing Jews, Muslims and some minor religious groups from the analysis.

Third we examined how key climate change beliefs differed across religious groups. These beliefs were whether or not: climate change was occurring, climate change was caused by humans, climate change was of personal importance, one’s own mitigation actions were effective, efforts to reduce global warming (given associated costs) were desirable and Australia should act to reduce climate change emissions before other larger developed and developing countries have begun to do so.

Previous research has shown that associations between religious affiliation and environmental attitude can be spurious (e.g. [14, 17]) and simply reflect other socio-demographic and attitudinal variables. To correct for this possibility and to isolate the effect of religion on segment membership, we ran a series of ordered logit regressions that predict segment membership on the basis of religion, as well as key socio-demographic and attitudinal variables. The ordered logit model is commonly specified as follows:

$$\begin{array}{l} yi*=\beta 'xi+\varepsilon i=\\ \begin{array}{l} wherey_{i}\;\;\;\;\;\;\;\;\;=\;0\;ify_{i}<{µ}_{0}\\ {}_{\;\;\;\;\;\;\;\;\;\;\;\;\;\;\;\;\;\;\;\;\;\;\;\;\;\;\;\;\;\;\;}=\;1\;if\;{µ}_{0}<y_{i}\;<\;{µ}_{1},\\ {}_{\;\;\;\;\;\;\;\;\;\;\;\;\;\;\;\;\;\;\;\;\;\;\;\;\;\;\;\;\;\;\;}=\;2\;if\;{µ}_{1}<y_{i}\;\;{µ}_{2},\\ \;\;\;\;\;\;\;\;\;\;\;\;\;\;\;\;\;\;\;\;\;\;\;\ldots \\ \;\;\;\;\;\;\;\;\;\;\;\;\;\;\;\;\;\;\;\;\;\;\;=\;J\;ify_{i}>\;{µ}_{J-1} \end{array} \end{array}$$

The free parameters μ_i_ represent the cut-off between ranks. Further details about this model can be found in Train [39].

Consistent with the previous literature that has found knowledge to be a predictor of environmental attitude (e.g. [21–23]), we included eight questions on climate change to provide an independent test of knowledge. This included questions such as “The hole in the ozone layer is a major climate change issue”, “Today, generating electricity from renewable sources (such as solar and wind) costs about the same as generating electricity from coal” and “The greenhouse effect is caused by heat from the sun being trapped by the atmosphere”. In addition, as climate change segment membership is likely to be influenced by environmental attitude, we also included in our questionnaire Dunlap et al.’s [40] revised New Environmental Paradigm (NEP) scale to measure respondents’ environmental attitudes. This 15-item scale has been previously found to include four factors [40].

## Results

The results on the association between religious affiliation and segment membership are presented in Table 2. The results suggest that the Christian non-literal denominations are likely to most closely represent the overall sample, though they do have a slightly smaller proportion in the *Alarmed* segment. The Atheist/Agnostic/No Religion group (43.8%) and Buddhists (45.7%) have a much higher percentage of people located in the *Alarmed* and *Concerned* segments than the sample overall (33.3%). Further, Atheist/Agnostic/No Religion (16.3%) and Buddhists (2.9%) have a lower percentage of people located in the *Doubtful* and *Dismissive* segments than the sample overall (20.6%). The respondents who indicate affiliation with Christian denominations that have a literal interpretation of scripture have a smaller proportion of people located in the *Alarmed* and *Concerned* segments (19.6%). The Christian literalists are also more likely to have a much higher percentage of people located in the *Doubtful* and *Dismissive* segments (30.2%) than the sample overall.

**Table 2:** Segment membership and religious affiliation.

	Alarmed	Concerned	Cautious	Disengaged	Doubtful	Dismissive	Total
Secularist	111	179	153	111	63	45	662
	(16.8%)	(27.0%)	(23.1%)	(16.8%)	(9.5%)	(6.8%)	
Buddhist	6	10	12	6	1	0	35
	(17.1%)	(28.6%)	(34.3%)	(17.1%)	(2.9%)	(0.0%)	
Christian—Literal	18	25	55	55	38	28	219
	(8.2%)	(11.4%)	(25.1%)	(25.1%)	(17.4%)	(12.8%)	
Christian—Non-literal	54	171	242	178	87	88	820
	(6.6%)	(20.9%)	(29.5%)	(21.7%)	(10.6%)	(10.7%)	
Overall Sample	10.8%	22.5%	26.1%	20.0%	11.3%	9.3%	

To further explore the relationship between religious affiliation and climate change beliefs, results are presented in Table 3 showing climate change beliefs for the four religious groups identified in this research. The results mostly mirror what was reported in Table 2. Those who identify with a Christian non-literal denomination have very similar attitudes to the sample overall. Those who are Buddhist or Atheist/Agnostic/No religion are more likely to believe: that global warming is occurring, in human causation, that there is a scientific consensus about global warming and the issue is important. They are also more likely to think that their own actions will make a difference, and that Australia should do something to reduce global warming, and to have a higher level of knowledge about climate change (as indicated by the test score). Again, those who identify with a Christian denomination with a literal view of scripture are less likely to believe: that global warming is occurring, in human causation, that there is a scientific consensus and that their own mitigation actions would be effective. They are also less likely to consider the issue to be important and that Australia should act to reduce emissions. ANOVA and Chi-square tests were also run to determine whether there were significant differences between the religious groups on these eight variables; there were in all cases (see final column of Table 3).

**Table 3:** Climate change beliefs across religious groups.

	Atheist/ Agnostic/ No religion	Buddhist	Christian—Literal	Christian—non Literal	Overall sample	F-Statistic/χ2
Certainty global warming is occurring^1^	7.02	7.63	5.79	6.3	6.54	24.357***
Believe that climate change is caused mostly by human activities	52.3%	77.1%	30.7%	41.8%	45.1%	52.525***
Believe that most scientists think global warming is happening	47.7%	51.1%	30.6%	34.0%	39.4%	38.457***
Perceived impact of own mitigation actions^2^	2.26	2.66	2.09	2.15	2.19	6.540***
Personal importance of issue^3^	3.11	3.37	2.77	2.87	2.96	10.659***
Desired Australian efforts to reduce warming given associated costs^4^	2.89	3.06	2.6	2.71	2.76	8.674***
Contingent international conditions for Australian mitigation action^5^	3.5	3.39	3.22	3.25	3.33	8.014***
Test result	3.97	4.06	3.58	3.41	3.72	10.117***

^1^ Certainty global warming is occurring is measured on a nine-point scale;

^2^ 1 = not at all, 4 = a lot;

^3^ 1 = not at all important, 5 = extremely important;

^4^ 1 = no effort, 4 = large-scale effort, even if it has economic consequences;

^5^ 1-Australia should not reduce its emissions, 4-Regardless of what other countries do Australia should reduce its emissions.

***Significant at 1%

The findings from this table and the previous one confirm that climate change beliefs are different across different religious groups. This raises the question of whether these differences are due to the religious beliefs of respondents, or whether they are due to attitudes, knowledge or socio-demographics, a question which we seek to answer next.

Before presenting the results of the regression analyses, we first present the results from a factor analysis conducted on the items in the NEP scale. As shown in the Rotated Component Matrix (see Table 4), we identified four factors which we labelled *Eco Crisis*, *Human Ingenuity*, *Human Rule* and *Earth Limits* based on the variables that loaded highly on each factor. The four factor solution had moderate total variance explained (58.6%), though a high KMO statistic (0.889). The reliability of two of the items was satisfactory, but the alphas for *Human Rule* and *Earth Limits* were slightly but not substantially below the standard acceptable level of 0.60.

**Table 4:** Rotated component matrix from the factor analysis, plus Cronbach Alpha (reliability) for each construct.

Scale Items	Eco Crisis	Human Ingenuity	Human Rule	Earth Limits
Humans are severely abusing the environment.	.770			
When humans interfere with nature, it often produces disastrous consequences.	.743			
If things continue on their present course, we will soon experience a major ecological disaster.	.676			
The balance of nature is very delicate and easily upset.	.641			
The earth has plenty of natural resources if we just learn how to develop them.		.731		
Human ingenuity will ensure that we do NOT make the earth unlivable.		.635		
The balance of nature is strong enough to cope with the impacts of modern industrial nations.		.538		
Human destruction of the natural environment has been greatly exaggerated.		.504		
Humans were meant to rule over the rest of nature.			.698	
Plants and animals have as much right as humans to exist.			-.641	
Humans have the right to modify the natural environment to suit their needs.			.637	
The earth has only limited room and resources.				.757
Despite our special abilities humans are still subject to the laws of nature.				.670
We are approaching the limit of the number of people the earth can support.				.614
Cronbach Alpha	0.762	0.695	0.548	0.580

Next we examined how the mean values for the four factor scores representing the four NEP sub-scales differ across the four religious groupings (see Table 5). One-way ANOVA’s indicate that there are significant differences only for three of the four variables: *Human Ingenuity*, *Human Rule* and *Earth Limits*. Christian literalists and non-literalists have a higher belief in the ability of human ingenuity to solve environmental problems than either Buddhists or Atheists/Agnostics or those with no religion. Interestingly, the highest levels of belief in human rule over nature are among Christian literalists and Buddhists, with Christian non-literalists having a fairly neutral value and Atheists/Agnostics/No Religion having a negative value. For belief in earth limits, the highest positive value was among those who are Atheists/Agnostics and those with no religion, Christian non-literalists again have a fairly neutral value, while Christian literalists and Buddhists again share a moderate to large negative value. At least in terms of belief in human rule and earth limits, Buddhist attitudes are similar to Christian ones, and both are quite different from Atheists/Agnostics/No Religion, as has been found by Hayes and Marangudakis [23].

**Table 5:** Climate change beliefs across religious groups.

	Atheist/ Agnostic/ No religion	Buddhist	Christian—Literal	Christian—non Literal	F-Statistic/χ2
Eco-crisis	0.04	0.10	-0.05	-0.05	1.124
Human ingenuity	-0.17	-0.31	0.08	0.11	10.961***
Human rule	-0.15	0.31	0.41	-0.02	19.509***
Earth limits	0.15	-0.24	-0.16	-0.04	8.223***

***Significant at 1%

Table 6 presents the results of three regression analyses that show the effect of religious affiliation, socio-demographics and environmental attitude, and knowledge on climate change segment membership. The *Alarmed* segment was scaled as “1” for the dependent variable and the *Dismissive* segment was scaled as “6”. The Atheist/Agnostic/No Religion category was omitted and the various effects were measured relative to this group. The first model only included religious affiliation as an independent variable. In this regression, similar to the results presented in Table 2, the Buddhist category was negatively signed but with an insignificant coefficient. The Christian non-literalist and Christian literalist had positively signed coefficients, indicating that members of both of these groups were less likely to be in one of the *Alarmed* or *Concerned* segments, and more likely to be in the *Doubtful* or *Dismissive* segments. Furthermore, the magnitude of the coefficient for the Christian literalists was almost one, implying that Christian literalists were on average likely to be located one segment lower than Atheist/Agnostic/No Religion respondents.

**Table 6:** Ordinal regressions showing the effect of religious variables, attitudes and socio-demographics on climate change segment membership.

	Model 1: Religious Variables Only	Model 2: Religious Variables Plus Attitudes and Socio-demographics	Model 3: Religious Variables Plus Attitudes and Socio-demographics Plus Interactions
*Religious Variables*			
Buddhist	-0.325 (1.102)	-0.634** (0.329)	-0.627* (0.343)
Christian Literal	0.988*** (0.140)	0.597*** (0.149)	0.509*** (0.154)
Christian Non-literal	0.597*** (0.094)	0.257*** (0.099)	0.244*** (0.099)
*Socio-demographic Variables*			
Gender (1 = Male)		0.498*** (0.101)	0.507*** (0.101)
Education (1-Never went to school, 11 = postgraduate qualification)		-0.093*** (0.025)	-0.094*** (0.025)
Full-time employed		0.307** (0.132)	0.304** (0.132)
Part-time employed		0.300** (0.152)	0.284* (0.153)
Self employed		0.536*** (0.210)	0.551*** (0.210)
Retirees		0.463*** (0.147)	0.451*** (0.147)
Test result		-0.189*** (0.030)	-0.189*** (0.030)
*Attitudinal Variables and Interactions*			
Eco crisis		-1.344*** (0.055)	-1.342*** (0.055)
Human ingenuity		0.867*** (0.049)	0.868*** (0.049)
Human rule		0.417*** (0.047)	0.530*** (0.080)
Earth limits		-0.369*** (0.047)	-0.379*** (0.048)
Human rule * Buddhist			-0.178 (0.287)
Human rule * Christian literal			0.063 (0.142)
Human rule * Christian non-literal			-0.229** (0.102)
McFadden R^2^	0.012	0.205	0.207

# The missing category for the religious variables is Atheist/Agnostic/No Religion; standard errors are in brackets;

*** significant at 1%,

** significant at 5%,

* significant at 10% level.

The dependent variable in all regressions is segment membership.

Overall this first regression had quite low explanatory power, with a McFadden R^2^ of 0.012. However, this score is different from an R^2^ in standard regression and is a relative measure that is comparable only to other models estimated on the same data set. Comparing the R^2^ of this model with the other two models, it is apparent that the explanatory power of models 2 and 3 is an order of magnitude better. This implies that religious affiliation had some explanatory power in terms of climate change beliefs, but that variables related to socio-demographics and attitudes collectively had much more explanatory power.

In Model 2 socio-demographic, attitudinal variables and knowledge (as represented by the test score) were included as explanatory variables in addition to religious affiliation. Being female, being more educated, not working or being self-employed or being retired, and knowledge of climate change were associated with a greater likelihood of being in a segment with greater concern about climate change. In terms of attitudes, concern about an ecological crisis or that the earth is reaching its limits was also associated with a greater likelihood of being in a segment with greater concern about climate change. However, human rule over nature and human ingenuity were associated with a reduced likelihood of being in a segment with greater concern about climate change. In this model the coefficient for Buddhism was significant, implying that once knowledge, socio-demographics and environmental attitude were taken into account, there was a difference between the Buddhist and the Atheist/Agnostic/No Religion categories in terms of their climate change attitudes and behaviors. The magnitude of the Christian non-literal and literal coefficients were substantially lower than in the previous model, but still showed moderate and significant effects. The signs of these various significant religion variables showed that the Buddhists were more concerned than the Atheist/Agnostic/No Religion group, and the two Christian groups less concerned. The magnitude of the coefficients for religious affiliation can be compared to other dummy variables to show relative effects. Thus the effect of being Buddhist or Christian literalist is similar in magnitude to the effect of gender and being self-employed, and larger than the effect of being employed full or part-time.

In Model 3 we present one additional regression where we included interactions between the religion variables and the variable representing human rule over nature. This interaction has been included given White’s [5] contention that there is a link between a Judeo-Christian perspective and a desire for dominion over nature. While no significant relationship was observed in the interaction term for either Buddhists or Christian literalists, intriguingly there was a negative and significant relationship between human rule and Christian non-literal, with the missing category being Atheist/Agnostic/No Religion. This relationship suggests that while human rule over nature does affect segment membership, the effect of a unit change in human rule over nature is smaller for Christian non-literalists than for those who are Atheist/Agnostic/No Religion. In addition, as shown in Table 5, Christian non-literalists had on average neutral views about human rule over nature, with Atheist/Agnostic/No Religion on average disagreeing with human rule over nature and Christian literalists and Buddhists agreeing on average. Taken together, these findings suggest that belief in human rule over nature affected segment membership; however the effect differed across religious groupings. The combined effect (beta weight times the mean value of the variable) was greatest for Christian literalists and Buddhists, followed by Christian non-literalists and then those who identified as Atheist/Agnostic/No Religion. These findings imply that White’s [5] contention that there is a link between a Judeo-Christian perspective and a desire for dominion over nature, and its consequences is more subtle than previously thought. It suggests that it is (1) not limited to a Judeo-Christian perspective, and (2) it differs in effect between Christian literalists and non-literalists.

## Discussion

There has been ongoing research about the relationship between religious beliefs and environmental attitudes. However, less research has focused on the link between religious affiliation and climate change beliefs, particularly in Australia. The results from this study provide clear evidence that among the Australian population attitudes to climate change and climate change policy differ across religious groups. The raw results suggest that Buddhists and Atheists/Agnostics/No Religion are likely to agree that there is climate change, it is human induced and to be supportive of policy. The Christian literalists are likely to have opposing views to this, and the Christian non-literalists are likely to hold opinions between the two extremes. These findings are consistent with those of Hand and van Liere [6] and Guth et al. [7] from the US, which showed a similar distribution of support for environmental policy among US secularists, Christian literalists and Christian non-literalists. Our results extend those of Tranter [2] who found that in Australia, there was a stronger support base for environmental action among those following eastern religious practices. By identifying the climate change attitudes of Buddhists, a group not previously evaluated in these earlier studies, we have contributed knowledge to this area of study. Furthermore, our results showed that Buddhists, like those in the Atheists/Agnostics/No Religion group, have a stronger potential for engagement in environmental issues than other religious groups; whereas Tranter [2] showed that “the non-religious are pro-environmental to a greater extent than those with religious affiliations”. These findings provide new insights into the linkages between religious belief and climate change attitudes.

Earlier studies [14, 17] suggested that the effect of religious variables on environmental concern might simply reflect social, demographic and other factors such as political conservatism. Others [21–23] have suggested that scientific knowledge may similarly be a mediating influence between religious variables and environmental concern. For this reason, we ran a series of ordinal regressions to examine the effect of socio-demographic variables, environmental attitude and knowledge about climate change on segment membership. Our results indicate that for those who are Christian literalist or Christian non-literalist, the effect of their religious affiliation substantially diminishes with inclusion of socio-demographic variables, environmental attitudes and knowledge. Nevertheless, religious affiliation was still demonstrated to be a significant factor; Christians had a tendency to be more dismissive of climate change than secularists, while Buddhists had stronger engagement and support for climate change policy than secularists. For all three of these religious groups, the effects could not simply be explained by socio-demographic variables, environmental attitude or knowledge about climate change. There was a residual effect related to their religious beliefs. These findings contrast with the earlier literature [14, 17].

Our findings provide new evidence to suggest that the differences associated with religious affiliation may in part be due to beliefs around human rule over nature. However, these beliefs are more nuanced than suggested by White [5]. Buddhists shared a similar level of support for human rule over nature with Christian literalists, though Buddhists had a strong level of support for pro-environment policies unlike Christian literalists. There was also a significant association between belief in human rule over nature and identification as a Christian non-literalist.

Christian literalists are likely to have a high belief in both human ingenuity to overcome environmental problems and in human rule over nature, and a low belief in earth limits. This set of beliefs is consistent with and may sustain a weak belief in (or even denial of) climate change as a problem and, if there is a problem, a strong belief in human capacity to overcome it. In contrast, those in the Atheist/Agnostic/No Religion group have the opposite views across these three dimensions. They tend to have a strong belief that there is a climate change problem and a weak belief in human capacity to overcome it.

While significant effects were identified in this study for all religious groups relative to the non-religious group, it should be borne in mind that these effects vary in magnitude. For the Buddhists, the effects were relatively large, and positively related to climate change engagement. For the Christian literalists and Christian non-literalists the effects were negatively related to climate change engagement and were similar in magnitude and direction to the effects of a number of socio-demographic variables such as, being male or being self-employed.

It would have been useful to have results that showed climate change attitudes and behavior for Indigenous Australians and those of different ethnic background. Unfortunately limitations of sample size prevented this. Some comments with respect to gender and education are, however, possible. From a literal Christian perspective, these results also present some further interesting challenges. Although the results show that the overall view in these congregations would be to disagree that there is climate change and that it is human induced, and to be unsupportive of policy, women members and those who are more highly educated would have a tendency to the opposite views on these issues.

For those of a Christian literalist or Christian non-literalist affiliation, the question arises about what should be done about these findings which show disengagement of Christians with climate change issues. One response is for denominations to seek to influence their members to bring an ethical dimension to the climate change issue, as recommended by Posas [4]. Indeed there has been evidence of this type of activity, such as in the Anglican diocese of Canberra and Goulburn (Australia) which has developed an environmental policy and is seeking to reduce climate change emissions from church buildings, and if possible by 50% by 2020 [41]. On a far larger scale is the Pope’s 2015 encyclical in which it is predicted that “he will define climate change as a fundamentally moral and religious challenge to the world” [11]. Climate change seems be an area in a deep state of flux as far as many religious groups are concerned. It is interesting that, in accord with our results, these examples of change in the positions of church leadership are from non-literal Christianity. An important question in this changing environment is the response among literal Christian leaders in the face of future climate change challenges. In this context, the response of conservative Christian leaders in the US has been to avoid the climate change issue [15].

Another possible response to our results is to seek a unified position across religious groupings on climate change, to seek to change community attitudes. Examples of these included the 2007 and 2009 resolutions of the United Churches of Christ in the USA, which comprises predominantly Congregational churches [42], the resolution of Evangelical Christian Leaders in the USA [43], as well as the position statements of the World Council of Churches [44]. Similar statements have also been released in Australia. For example, the Uniting Church [45] adopted a statement called “For the Sake of the Planet and all its People” that relates to climate change, and calls on church members to reduce carbon emission and advocate for governments to reduce dependence on carbon based energy sources. Similar efforts are being promoted by the Catholic Church through Catholic Earthcare Australia [46]. Evidence is emerging that these sorts of strategies have led to increased local level action to reduce carbon emissions [47]. However, as Ayre [47] suggests, ambiguity still remains for some Australian denominations about the church’s role in being vocal about the need for responsible care for the environment, and opportunity remains to better translate stated positions about climate change into effective work on the ground. Thus there remains opportunity for religious groups to do more to support the acceptance of human induced climate change and the need for policy responses, as well as action at a local level.

## Conclusions

Based on data from an online survey of 1,927 Australians, we conducted various quantitative and qualitative analyses examining the relationship between religious affiliation and climate change attitudes. We found substantive effects on climate change beliefs based on religious affiliation. Christians (both literalists and non-literalists) were the least engaged with climate change issues and Buddhists the most engaged. For all of the religious groupings (Buddhist, Christian literalists and Christian non-literalists) the effects cannot be explained by socio-demographics, environmental attitudes, or environmental knowledge alone.

In terms of the five research questions behind the study, first, after accounting for socio-demographics, environmental attitudes and environmental knowledge, Buddhists were the group most engaged with climate change issues. Second, Christian literalists were the least engaged group and least likely to support climate change action. Christian non-literalists were also largely disengaged. To change their members’ perspective on climate change, leaders of these Christian denominations will need to develop an ethical dimension to the climate change issue.

Third, although the results indicate that knowledge of climate change issues is likely to increase engagement, the appropriate method of increasing knowledge is not clear. Simply providing scientific information about climate change has resulted in some perverse impacts on climate change beliefs [48]. Fourth, the results suggest that in Australia broad belief systems do form the basis of climate change attitudes and behaviors. Certain components of these belief systems resemble those in the US, for example, many Christian literalists seem to have a ‘development stewardship’ perspective [49] that is averse to policy intervention to combat climate change. Fifth, our results on beliefs about human rule over nature and its relationship to climate change attitudes were more nuanced than White’s [5] hypothesis suggests. Christian literalists and Buddhists had a stronger belief in human rule over nature than either Christian non-literalists or those in the Atheist/Agnostic/No Religion group. However, importantly, the human rule of Buddhists seems to be associated with support for the environment, whereas for Christian literalists it is not.

The next steps in this line of research would include replication of the research in other countries where climate change and related policy are likely to be significant issues, particularly in those countries with a different mix of religious groups; deepening our understanding of how membership of various religions influences climate change attitudes and behaviors; and better understanding of how to appeal to members of different religious groupings in order to modify their climate change attitudes and behaviors where needed.
